# Noncontrast cardiac computed tomography‐derived mitral annular calcification scores in mitral valve disease

**DOI:** 10.1002/clc.24110

**Published:** 2023-08-04

**Authors:** Jie Hou, Yu Sun, Huishan Wang, Libo Zhang, Jinglong Shi, Hongrui You, Rongrong Zhang, Benqiang Yang

**Affiliations:** ^1^ College of Medicine and Biological Information Engineering Northeastern University Shenyang Liaoning China; ^2^ Department of Radiology General Hospital of Northern Theater Command Shenyang Liaoning China; ^3^ Key Laboratory of Cardiovascular Imaging and Research of Liaoning Province Shenyang Liaoning China; ^4^ Department of Cardiovascular Surgery General Hospital of Northern Theater Command Shenyang Liaoning China

**Keywords:** arrhythmia, mitral annular calcification, mitral annular calcification score, mitral valve disease, noncontrast cardiac computed tomography

## Abstract

**Background and Aims:**

Mitral annular calcification (MAC) by computed tomography (CT) is reported as an independent predictor of poor outcomes. However, it currently remains unclear if quantitative MAC parameters provide more value for mitral valve disease (MVD) management, therefore, we examined the prognostic value of MAC scores using noncontrast cardiac‐CT in MVD patients.

**Methods:**

Between January 2020 and December 2021, we prospectively enrolled 300 consecutive patients with MVD (MAC‐present = 80 and MAC‐absent = 220) undergoing preoperative cardiac‐CT and mitral valve (MV) surgery. Noncontrast cardiac‐CT images were used to qualitatively detect MAC (present or absent) and evaluate MAC scores. For analyses, we also collected baseline clinical data, intraoperative conversion (from MV repair to MV replacement), and follow‐up arrhythmia data.

**Results:**

Compared with the MAC‐absent group, MAC‐present patients were older (62 ± 7 vs. 58 ± 9 years, *p* < .001), mostly women (55% vs. 39.5%, *p* = .017), and also had aortic valve calcification (57.5% vs. 23.2%, *p* < .001), mitral stenosis (82.5% vs. 61.8%, *p* < .001), atrial fibrillation (30% vs. 11.8%, *p* < .001), and larger left atrial end‐diastolic dimension (LADD, 49 [44–56] versus 46 [41–50], *p* = .001]. Furthermore, MAC‐present patients underwent more MV replacements (61.8% vs. 82.5%, *p* = .001) and experienced a higher intraoperative conversion prevalence (11.8% vs. 61.3%, *p* < .001). Multiple logistic regression analyses showed that the female gender (odds ratio [OR]/95% confidence interval [CI]/*p* = 2.001/1.042–3.841/0.037) and MAC scores (OR/95% CI/*p* = 10.153/4.434–23.253/*p* < .001) were independent predictors of intraoperative conversion. During a follow‐up of 263 ± 134 days, MAC‐present patients had more arrhythmias (42.5% vs. 9.5%, *p* < .001). Also, MAC‐scores (hazard ratio [HR]/95% CI/*p* = 6.841/3.322–14.089/*p* < .001) and LADD (HR/95% CI/*p* = 1.039/1.018–1.060/*p* < .001) were independently associated with arrhythmias by Cox regression analyses.

**Conclusions:**

Noncontrast cardiac CT‐derived MAC‐scores showed a high risk for intraoperative conversion and follow‐up arrhythmias in MVD‐patients.

## Graphical abstract

Mitral annular calcification (MAC) score: Distribution, measurement and prognosis. The present analysis is based on noncontrast cardiac computed tomography (CT) to semiautomatically quantitatively measure the MAC score using Agatston's method in mitral valve disease (MVD). Noncontrast cardiac CT‐derived MAC scores showed a high risk for intraoperative conversion and follow‐up arrhythmias in MVD patients. The annular segments: A1, A2, A3, segments of the anterior mitral annulus; P1, P2, P3, segments of the posterior mitral annulus, from lateral to medial.

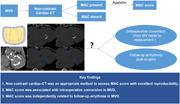

**Abbreviations**
AF: atrial fibrillation
AS: aortic stenosis
AVC: aortic valve calcification
CI: confidence interval
CT: computed tomography
ECG: electrocardiography
HR: hazard ratio
ICC: intraclass correlation coefficients
LADD: left atrial end‐diastolic dimension
LVOT: left ventricular outflow tract
MAC: mitral annular calcification
MDCT: multidetector computed tomography
MR: mitral regurgitation
MS: mitral stenosis
MV: mitral valve
MVD: mitral valve disease
OR: odds ratio
ROC: receiver operating characteristic

## 1 INTRODUCTION

Characterized as a progressive and chronic degenerative process in the fibrous annulus of the mitral valve (MV), mitral annular calcification (MAC) is often an incidental, asymptomatic, and under‐reported finding.^1^, ^2^ Typically affecting the posterior aspect of the annulus fibrosa, MAC may extend to the anterior aspect, involve the entire annular circumference or myocardium and mitral leaflets, and lead to MV dysfunction.^1^, ^2^, ^3^, ^4^ The condition is also associated with elevated left ventricular afterload, including hypertrophic cardiomyopathy with obstruction, hypertension, and valvular aortic stenosis (AS). MAC may lead to mitral stenosis (MS) and/or mitral regurgitation (MR), with concomitant severe AS requiring ameliorative double‐valve intervention.^5^, ^6^

MAC is associated with elevated perioperative complications and all‐cause mortality risks^1^, ^2^, ^5^; it reportedly causes a sixfold increase in operative mortality in patients undergoing isolated MV surgery,^7^ while early mortality rates, upon surgical MV replacement in MAC, reportedly as high as 28%.^1^ Generally, replacing or repairing MV in severely affected patients with MAC is technically difficult, even when concomitant aortic valve replacement risks are removed.^8^, ^9^

Multidetector computed tomography (MDCT) generates detailed MAC assessment before surgery and may alter therapeutic strategies.^10^ Noncontrast cardiac computed tomography (CT) is reported as viably assessing valve calcification scores.^11^, ^12^, ^13^ The approach, characterized by high X‐ray calcium attenuation, excellent spatial resolution, and three‐dimensional postprocessing analysis, assesses MAC characteristics, total calcium distribution, and coronary artery and aortic valve calcification (AVC).^10^, ^14^

MAC is reportedly an independent predictor of poor outcomes,^1^, ^2^, ^5^ with prognostic value for atrial fibrillation (AF) ablation^15^ and transcatheter aortic valve implantation.^3^ Noncontrast cardiac CT is a semi‐automated quantification method for calculating calcium burden (MAC scores), such as coronary calcification scores which are used to quantitatively evaluate calcification, and provide more accurate risk assessments and disease prognosis predictions for multicenter clinical research. However, the value of MAC scores from noncontrast cardiac CT for mitral valve diseases (MVDs) have been rarely reported. The literature is limited in determining if MAC is associated with surgical method choice and predicting postoperative arrhythmia in MVD. Therefore, new investigations must ascertain if MAC provides useful clinical information enabling early intervention and improving treatment strategies. In our retrospective study, we examined MAC incidence, explored the clinical value of MAC scores in selecting surgical methods, and identified its potential predictive power in patients with MVD.

## 2 PATIENTS AND METHODS

After approval from our ethics committee, informed patient consent was waived due to the retrospective nature of our investigation.

### 2.1 Patient selection

In our single‐center hospital (in the Department of Cardiovascular Surgery), between January 2020 and December 2021, we evaluated 300 consecutively admitted patients with MVD. We adhered to the 2017 European Society of Cardiology/European Association for Cardio‐Thoracic Surgery guidelines outlining MVD patient management.^16^ Upon admission and before surgery, patients underwent echocardiography and electrocardiography (ECG)/dynamic ECG.^17^ Before surgery, patients also underwent cardiac computed tomography angiography to evaluate coronary artery and intracardiac disease. To be included in the study, patients with MVD were ≥18 years or older, had noncontrast cardiac CT detection, and had undergone MV surgery. Patients were excluded if they had previous valvular surgery or ablation for AF, poor image quality, and had not undergone previous surgery. Patient basic characteristics, intraoperative conversion, follow‐up data, and arrhythmia information were retrospectively collected from the 300 patients.

### 2.2 Imaging

Imaging was performed using a 640‐slice MDCT scanner (Aquilion ONE Vision Edition; Canon Medical Systems Corporation) with prospective ECG gating. Imaging parameters included: tube voltage = 100 kV, tube current = SD32 mAs, field of view = 260 × 260 cm, slice thickness = 0.5 mm, matrix = 512 × 512, detector width = 16 cm, and reconstruction phase = 75%. ECG editing technology was used to reconstruct images in severe arrhythmia.

### 2.3 MAC

MAC was examined using noncontrast cardiac‐gated CT. MAC scores and distribution were gathered using the Agatston method^18^, ^19^ (Supporting Information: Figure S1). Noncontrast images (0.5‐mm slices and 0.5‐mm increments) were assessed using semiautomatic software (VScore, Vitrea, Vital Images). We selected the diastolic phase of the cardiac cycle, with maximal MV plane, using 4‐ and 2‐chamber views. We recorded calcific deposit status in MVs or annulus segments. Manual editing was performed to eliminate aortic or coronary calcium.

**Figure 1:**
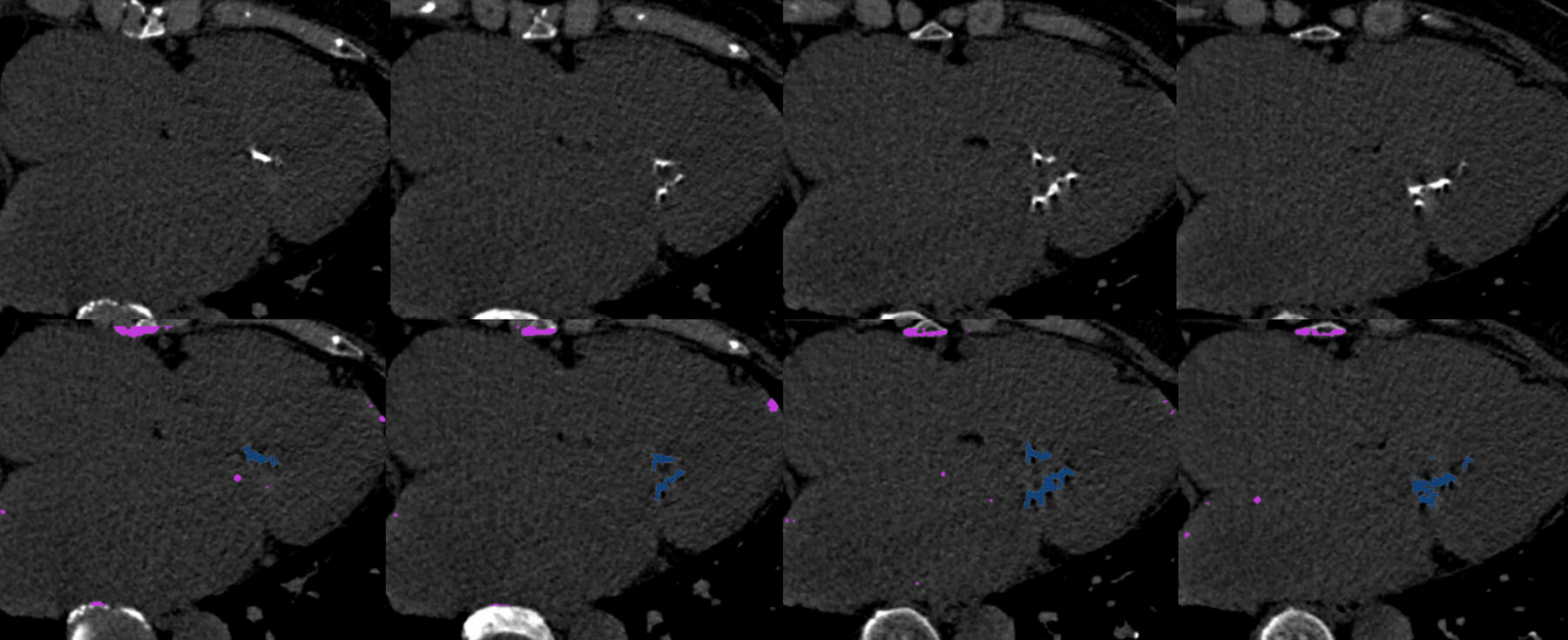
Multiplanar measurement of MAC. Figure 1 showed the case of a mitral valve disease with MAC. MAC score was evaluated by CT. MAC volume was measured at 1372 mm^3^, MAC score was measured at 1767. CT, computed tomography; MAC, mitral valve calcification.

To define calcium areas, Agatston scores using a CT attenuation threshold = 130 Hounsfield units were used,^12^ and the maximum CT attenuation in lesions was used to generate weighting scores. Weight = 1 indicated an attenuation of 130–199; 2 = 200–299; 3 = 300–399; and 4 ≥ 400.^13^ The weighting factor was multiplied by lesion area, with the total of lesions values used to determine total Agatston scores^13^, ^20^ (Figure 1). CT scans were separately and independently analyzed by two experienced and blinded cardioradiologists (≥5 and ≥3 years' experience, respectively). Discordance was settled by discussion and consensus. MAC scores were also recorded by cardioradiologists to identify intraclass correlation coefficients (ICCs) and evaluate the precision and accuracy of the MAC score method.

### 2.4 Patient follow‐up

Patients were followed up to December 2022. Primary study outcomes were arrhythmia (including atrioventricular block, AF, and bundle branch block) recurrence during routine follow‐up (>3 months postsurgery), which required direct or drug current cardioversion. In the first 3 months postsurgery (“blank period”), arrhythmias were not recorded as adverse events. The period between the surgery date and arrhythmia recurrence was recorded as the time to event (arrhythmia recurrence). Follow‐up at outpatient visits or rehospitalization included echocardiography and ECG evaluations after surgery.

### 2.5 Statistical analyses

Data were analyzed in SPSS v. 20.0 (IBM Corporation). Continuous data were represented as the mean ± standard deviation or median (quartiles), and analyzed using independent sample *t*‐ or Mann–Whitney *U* tests. Normal distributions across continuous variables were examined using Kolmogorov–Smirnov tests. Categorical data (numbers and percentages) were analyzed using Fisher's exact or Pearson's *χ* ^2^ tests (Tables 1 and 2). Inter‐ and intraobserver agreement data for subjectively assessing MAC and AVC occurrence were evaluated using cross‐tabulation and kappa (*κ*) calculations. To determine significant independent predictors, multivariate logistic regression analyses were performed (Table 3). Also, to determine collinear covariates, multicollinearity analyses were performed. We used Cox regression for follow‐up arrhythmia analyses after surgery, and parameters with significant effects in univariate Cox regression analysis underwent multivariate Cox regression (Table 4). We used receiver operating characteristic (ROC) analyses to examine predictive potential factors in a multivariate‐adjusted logistic regression model. A *p* < .05 value indicated statistical significance.

**Table 1:** Clinical characteristics of all patients.

Variable	All patients (*n* = 300)	Patients grouped by MAC		*p*‐Value
		Present (*n* = 80)	Absent (*n* = 220)	
Clinical characteristics				
Age (mean ± SD, years)	59 ± 9	62 ± 7	58 ± 9	<.001
Female, *n* (%)	131 (43.7)	44 (55)	87 (39.5)	.017
BMI (kg/m^2^)	24 ± 3.5	24 ± 3.8	24 ± 3.5	.981
Heart rate (bpm)	84 ± 14	85 ± 17	83 ± 12	.383
Chronic kidney disease, *n* (%)	3 (1)	0 (0)	3 (1.4)	.567
Hypertension, *n* (%)	168 (56)	35 (43.8)	133 (60.5)	.010
Diabetes mellitus, *n* (%)	22 (7.3)	8 (10)	14 (6.4)	.285
Smoking, *n* (%)	79 (26.3)	20 (25)	59 (26.8)	.752
Alcohol, *n* (%)	75 (25)	16 (20)	59 (26.8)	.228
Prior stroke/TIA history, *n* (%)	15 (5)	4 (5.0)	11 (5.0)	1.000
Coronary heart disease, *n* (%)	82 (27.3)	22 (27.5)	60 (27.2)	.969
Paroxysmal or chronic AF, *n* (%)	50 (16.7)	24 (30)	26 (11.8)	<.001
Conduction system disease, *n* (%)	18 (6)	5 (6.3)	13 (5.9)	1.000
NYHA class on admission ≥ III, *n* (%)	293 (97.7)	80 (100)	213 (96.8)	.237
NT‐ProBNP (pg/mL, median [IQR])	604 (184–1584)	836 (326–1669)	548 (167–1504)	.032
MAC score		911.6 ± 1852	–	–
AVC present on CT, *n* (%)	97 (32.3)	46 (57.5)	51 (23.2)	<.001
Echocardiography on admission				
LADD (mm, median [IQR])	47 (42–51)	49 (44–56)	46 (41–50)	0.001
LVEDV (mL)	141 ± 56	127 ± 53	146 ± 56	0.008
LVESV (mL)	62 ± 31	57 ± 29	64 ± 31	0.076
LVEF (%)	56.4 ± 5.3	55.7 ± 4.8	56.7 ± 5.5	0.166
Aortic stenosis, *n* (%)	56 (18.7)	27 (33.8)	29 (13.2)	<0.001
Aortic regurgitation, *n* (%)	91 (30.3)	33 (41.3)	58 (26.4)	0.013
Mitral stenosis, *n* (%)	73 (24.3)	43 (53.8)	30 (13.6)	<0.001
Mitral regurgitation, *n* (%)	300 (100)	80 (100)	220 (100)	–
Tricuspid regurgitation, *n* (%)	186 (62)	55 (68.8)	131 (59.5)	.146
Mitral valve prolapses	161 (53.7)	15 (18.8)	146 (66.4)	<.001
Surgery, *n* (%)				
Mitral valve repair	98 (32.7)	14 (17.5)	84 (38.2)	.001
Mitral valve replacement	202 (67.3)	66 (82.5)	136 (61.8)	.001
Aortic valve replacement	73 (24.3)	26 (32.5)	47 (21.4)	.047
Tricuspid valve repair	151 (50.3)	49 (61.3)	102 (46.4)	.023
Tricuspid valve replacement	2 (0.7)	1 (1.3)	1 (0.5)	.463
Coronary artery bypass grafting	29 (9.7)	8 (10)	21 (9.5)	.906
Maze procedure	15 (5)	6 (7.5)	9 (4.1)	.369
Intraoperative conversion	75 (25)	49 (61.3)	26 (11.8)	<.001
Followed‐up echocardiography				
LADD (mm, median [IQR])	40 (38–44)	43 (40–51)^a^	40 (37–42)^b^	<.001
LVEDV (mL)	100 ± 29	99 ± 24^a^	101 ± 30^b^	.531
LVESV (mL)	45 ± 16	44 ± 14^a^	45 ± 17^b^	.646
LVEF (%)	56 ± 4.3	56.1 ± 4.2	56.1 ± 4.3^b^	.505
Admission time (day)	19.8 ± 6.5	19.3 ± 7.1	19.9 ± 6.3	.508
Follow‐up time (day)	263 ± 134	242 ± 104	268 ± 143	.145
Arrhythmia, *n* (%)	55 (18.3)	34 (42.5)	21 (9.5)	<.001

*Note*: “a”/“b” indicates statistical significance between the echocardiographic findings on admission and the followed‐up findings in MAC‐present/MAC‐absent group.

Abbreviations: AF, atrial fibrillation; AVC, aortic valve calcification; BMI, body mass index; IQR, interquartile range; LADD, left atrial end‐diastolic dimension; LVEDV/LVESV, left ventricular end‐diastolic/end‐systolic volume; LVEF, left ventricle ejection fraction; MAC, mitral annular calcification; *n*, number; NYHA, New York Heart Association; SD, standard deviation; TIA, transient ischemic attack.

**Table 2:** Clinical characteristics of patients with intraoperative conversion from mitral valve repair to replacement.

Variable	Intraoperative conversion		*p*‐Value
	With (*n* = 75)	Without (*n* = 225)	
Clinical characteristics			
Age (mean ± SD, years)	59.6 ± 9.94	59.4 ± 8.72	.296
Female, *n* (%)	44 (58.7)	87 (38.7)	.002
BMI (kg/m^2^)	23.9 ± 3.86	24.1 ± 3.44	.590
Hypertension, *n* (%)	38 (50.7)	130 (57.8)	.283
Diabetes mellitus, *n* (%)	8 (10.7)	14 (6.2)	.201
Smoking, *n* (%)	18 (24)	61 (27.1)	.596
Alcohol, *n* (%)	14 (18.7)	61 (27.1)	.144
Prior stroke/TIA history, *n* (%)	4 (5.3)	11 (4.9)	1.000
Coronary heart disease, *n* (%)	20 (26.7)	62 (27.6)	.881
Paroxysmal or chronic AF, *n* (%)	22 (29.3)	28 (12.4)	.001
Conduction system disease, *n* (%)	7 (9.3)	11 (4.9)	.261
NT‐ProBNP (pg/mL, median [IQR])	936.5 (423.5–1827)	547 (174–1497.5)	.008
MAC present on CT, *n* (%)	49 (65.3)	31 (13.8)	<.001
MAC score	324.3 ± 889.1	183.5 ± 957.8	.003
AVC present on CT, *n* (%)	29 (38.7)	68 (30.2)	.176
Echocardiography on admission			
LADD (mm, median [IQR])	48 (45–56)	40 (37–43)	.001
LVEDV (mL)	127.5 ± 52.7	145.4 ± 56.1	.016
LVESV (mL)	57.2 ± 28.9	63.7 ± 31.4	.117
LVEF (%)	55.8 ± 5.04	56.7 ± 5.40	.237
Aortic stenosis, *n* (%)	21 (28)	35 (15.6)	.017
Aortic regurgitation, *n* (%)	27 (36)	64 (28.4)	.218
Mitral stenosis, *n* (%)	35 (46.7)	38 (16.9)	<.001
Mitral regurgitation, *n* (%)	75 (100)	225 (100)	–
Tricuspid regurgitation, *n* (%)	55 (73.3)	131 (58.2)	.020

Abbreviations: AF, atrial fibrillation; AVC, aortic valve calcification; BMI, body mass index; IQR, interquartile range; LADD, left atrial end‐diastolic dimension; LVEDV/LVESV, left ventricular end‐diastolic/end‐systolic volume; LVEF, left ventricle ejection fraction; MAC, mitral annular calcification; *n*, number; NYHA, New York Heart Association; SD, standard deviation; TIA, transient ischemic attack.

**Table 3:** Factors associated with intraoperative conversion from mitral valve repair to replacement.

Variables	VIF	Univariable analysis		Multivariable analysis	
		OR (95% CI)	*p*‐Value	OR (95% CI)	*p*‐Value
Female	1.124	2.251 (1.322, 3.833)	.003	2.040 (1.061, 3.924)	.033
AF	1.305	2.043 (1.113, 3.749)	.021	0.764 (0.319, 1.830)	.546
MAC score^c^	1.239		<.001		<.001
>268		13.786 (6.396, 29.714)	<.001	10.153 (4.434, 23.253)	<.001
≤268		10.095 (4.775, 21.343)	<.001	7.942 (3.575, 17.642)	<.001
LADD	1.360	1.053 (1.021, 1.086)	.001	1.034 (0.993, 1.077)	.108
AS	1.149	2.111 (1.136, 3.923)	.018	0.973 (0.452, 2.093)	.943
MS	1.448	4.306 (2.430, 7.631)	<.001	1.479 (0.679, 3.220)	.325

*Note*: “c” indicates that patients were divided into negative MAC (MAC score = 0, *n* = 220), lower MAC score (0 < MAC score ≤ 268, *n* = 40) and higher MAC score (MAC score > 268, *n* = 40) subgroups.

Abbreviations: AF, atrial fibrillation; AS, aortic stenosis; CI, confidence interval; LADD, left atrial end‐diastolic dimension; MAC, mitral annular calcification; MS, mitral stenosis; OR, odds ratio; VIF, variance inflation factor.

**Table 4:** Predictors of follow‐up arrythmia with Cox regression analysis in patients with MVD.

Variables	Univariable analysis		Multivariable analysis	
	HR (95% CI)	*p*‐Value	HR (95% CI)	*p*‐Value
Hypertension	0.573 (0.334, 0.984)	.043	0.966 (0.533, 1.752)	.910
MAC score^c^		<.001		<.001
>268	7.948 (4.382, 14.415)	<.001	6.897 (3.349, 14.207)	<.001
≤268	2.653 (1.202, 5.858)	.016	2.952 (1.289, 6.764)	.010
LADD	1.057 (1.039, 1.075)	<.001	1.038 (1.018, 1.059)	<.001
AS	2.308 (1.313, 4.057)	.004	1.303 (0.703, 2.417)	.400
MS	1.734 (1.012, 2.972)	.045	0.518 (0.266, 1.011)	.054

*Note*: “c” indicates that patients were divided into negative MAC (MAC score = 0, *n* = 220), lower MAC score (0 < MAC score ≤ 268, *n* = 40) and higher MAC score (MAC score > 268, *n* = 40) subgroups.

Abbreviations: AF, atrial fibrillation; AS, aortic stenosis; CI, confidence interval; HR, hazard ratio; LADD, left atrial end‐diastolic dimension; MAC, mitral annular calcification; MS, mitral stenosis; MVD, mitral valve disease.

## 3 RESULTS

### 3.1 Study population

Of the 300 patients (mean age = 59 ± 9 years and 43.7% were females), 80/300 (26.7%) were assigned to the MAC‐present group and 220/300 (73.3%) to the MAC‐absent group. When compared with the MAC‐absent group, patients with MAC were older (58 ± 9 vs. 62 ± 7 years, *t* = 3.353, *p* < .001), mainly female (39.5% vs. 55%, *χ* ^2^ = 5.696, *p* = .017), and had hypertension (*χ* ^2^ = 13.964, *p* < .001), paroxysmal or chronic AF (*χ* ^2^ = 13.964, *p* < .001), AVC (*χ* ^2^ = 31.58, *p* < .001), AS (*χ* ^2^ = 16.347, *p* < .001), aortic regurgitation (AR, *χ* ^2^ = 6.152, *p* = .013), MS (*χ* ^2^ = 51.271, *p* < .001), and larger left atrial end‐diastolic dimension (LADD) at admission (*Z* = −3.336, *p* = .001).

From surgery data, MV replacement (*χ* ^2^ = 11.409, *p* = .001), aortic valve replacement (*χ* ^2^ = 3.952, *p* = .047), and intraoperative conversion from MV repair to replacement ratios were higher in the MAC‐present group when compared with the MAC‐absent group (*χ* ^2^ = 76.455, *p* < .001). No significant differences (*p* > .05) in follow‐up duration were observed between groups. During follow‐up (263 ± 134 days), patients with MAC had a higher arrhythmia prevalence (*χ* ^2^ = 42.554, *p* < .001), while follow‐up echocardiographic findings were significantly improved when compared with those at admission (*p* < .05). Additionally, a significant difference was observed in follow‐up LADD between groups after follow‐up (*Z* = −5.051, *p* < .001; Table 1).

### 3.2 MV calcification assessment

We identified 80 patients (26.7%) with MAC and observed that calcific deposits were more frequent on the posterior mitral annulus when compared with the anterior. MAC location: A1/A2/A3/P1/P2/P3 = 27 (33.8%)/20 (25%)/28 (35%)/44 (55%)/34 (42.5%)/30 (37.5%). MAC thickness = 4.1 ± 2.5 mm, MAC volume = 703 ± 1348 mm^3^, left ventricular outflow tract (LVOT) calcification was identified in 25 cases (25/80, 31.3%), and LVOT calcification volume = 447 ± 1293 mm^3^. In patients with MAC, the mean score of MAC was 911.6 ± 1852. Excellent intra‐ (*κ* = 0.98) and interobserver (*κ* = 0.97) agreement scores were recorded between operators assessing MAC on the same noncontrast cardiac‐gated CT images. Patients with MAC had a higher MS incidence when compared with patients without MAC (53.8% vs. 13.6%, *p* < .001).

### 3.3 Reproducibility of MAC scores

Intra‐ and interobserver MAC‐score reproducibility was examined using semiquantitative analyses. Excellent intra‐ (ICC = .998; .998–.999) and interobserver reproducibility (ICC = .996; .995–.997) scores were recorded.

### 3.4 Factors related to intraoperative conversion from MV repair to replacement

Patients were classified into two groups: those with intraoperative (75/300, 25%) and those without intraoperative conversion (225/300, 75%). Intraoperative conversion was more prevalent in females (58.7% vs. 38.7%, *χ* ^2^ = 9.147, *p* = .002), and patients had a greater incidence of paroxysmal or chronic AF (29.3% vs. 12.4%, *χ* ^2^ = 11.552, *p* = .001), AS (28% vs. 15.6%, *χ* ^2^ = 5.738, *p* = .017), MS (46.7% vs. 16.9%, *χ* ^2^ = 27.089, *p* < .001), MAC (65.3% vs. 13.8%, *χ* ^2^ = 76.455, *p* < .001), higher MAC scores (324.3 ± 889.1 vs. 183.5 ± 957.8, *Z* = − 2.951, *p* = .003), and larger LADD (48 [45–56] versus 40 [37–43], *Z* = − 3.339, *p* = .001) (Table 2). Univariate logistic regression analyses indicated that female gender, AF, MAC, MAC scores, LADD, AS, and MS had significant associations with intraoperative conversion in binary analyses. Multiple logistic regression analyses identified female gender (odds ratio [OR] = 2.001; 95% confidence interval [CI]: 1.042–3.841; *p* = .037) and MAC scores (OR = 10.153; 95% CI: 4.434–23.253; *p* < .001) as independently associated with intraoperative conversion after adjusting for AF, LADD, AS, and MS (Table 3). ROC curves showed that MAC scores exhibited a relatively good capacity in predicting intraoperative conversion (area under the ROC curve [AUC] = 0.76; 95% CI: 0.692–0.831; *p* < .001), followed closely by female gender (AUC = 0.6; 95% CI: 0.526–0.674; *p* = .009).

### 3.5 Follow‐up arrhythmias

In total, 55 (55/300, 18.3%) patients had follow‐up arrhythmias (including AF, atrioventricular block, and bundle branch block) postsurgery, over a 219 ± 133 days follow‐up. Using univariate Cox regression analyses, hypertension, MAC scores, LADD, AS, and MS were univariate predictors of recurrent arrhythmias in patients with MVD postsurgery. Multivariate Cox regression analyses indicated that MAC scores (hazard ratio [HR] = 6.841; 95% CI: 3.322–14.089; *p* < .001) and LADD (HR = 1.039; 95% CI: 1.018–1.060; *p* < .001) remained significant (Table 4). ROC curves showed that LADD had a relatively good capacity in predicting arrhythmia (AUC = 0.772; 95% CI: 0.706–0.837; *p* < .001), followed closely by MAC scores (AUC = 0.739; 95% CI: 0.656–0.822; *p* < .001).

## 4 DISCUSSION

When compared with the MAC‐absent group, patients with MAC were older and mainly female, and had AF, AVC, AS, MS, and larger LADD. Patients with MAC had a higher prevalence of intraoperative conversion from MV repair to replacement. MAC scores and female gender were independent predictors of intraoperative conversion. During follow‐up, MAC patients had an increased arrhythmia incidence. MAC scores and LADD were independent arrhythmia predictors. Thus, MAC was an important imaging index in MVD prognosis outcomes and treatment. MAC scores, based on quantitative nonenhanced cardiac CT evaluations, were important in predicting intraoperative conversion and postoperative arrhythmia events in patients with MVD.

Recent studies reported that MAC is an active and controlled molecular event associated with microscopic and macroscopic injury, lipid deposition, hemodynamic stress, chronic kidney disease, dysregulated bone and mineral metabolism regulators, and local inflammation.^21^, ^22^ Baseline MAC burden was also related to disease activity and disease progression rates.^21^ MAC appears to induce anatomical changes which culminate in either MS or combined MS and MR, while MS in severe MAC settings is caused by encroaching orifice areas, and rheumatic MS arises due to an absence of leaflet commissural union.^23^ MR is generated by an altered annulus during systole or leaflet coaptation distortion, which cause left atrium volume and pressure overload, leading to enlargement.^24^, ^25^ Pawade et al.^13^ reported that AVC should be measured using noncontrast CT and the Agatston approach. In the valve, the majority of data are related to Agatston scores and not calcium volume measurements. Density weighting is likely advantageous, the denser the calcium deposits, the more likely they will cause hemodynamic obstruction and valve‐leaflet stiffening. We recorded excellent inter‐ and intraobserver agreements between operators who measured MAC from cardiac CT images, consistent with previous studies.^2^, ^26^ We showed that noncontrast cardiac‐gated CT is a good semiquantitative method assessing MAC severity. MAC is common in cardiovascular imaging and postmortem and surgical samples, with an estimated 8%–42% prevalence.^11^ We also showed that MAC prevalence in patients with MVD was 25.9%, consistent with previous results.^11^ Patients with MAC were advanced in age and more likely to be female, with hypertension and valvular heart disease. Critically, similar results were reported in previous studies.^27^, ^28^, ^29^ MAC was also associated with cardiovascular risk factors.^27^, ^28^, ^29^ These observations suggested overlapping but distinct mechanisms underlying these pathologies.

Interestingly, MAC scores and female gender were independent risk markers for intraoperative conversion; indeed, the literature indicated that MAC was more prevalent in females.^21^, ^30^ While surgical treatment in patients with MAC is technically complex, there is a need for annular reconstruction and adequate debridement before MV replacement or repair.^31^ In such cases, MV repair may not be undertaken due to difficulties suturing calcified sites and severe calcification, thereby requiring prosthetic valve replacement. Patients with MAC experience significant comorbidities and have worse survival outcomes, although MAC is not a mortality risk factor.^32^ MAC alone, irrespective of severity, is independently related to adverse postoperative outcomes and elevated operative mortality.^33^ Potentially, MAC may provide preoperative evaluations, with MAC scores routinely incorporated as integral to prevalve surgery evaluations.

Additionally, MAC and LADD were independent risk markers for follow‐up arrhythmias. Framingham Heart and Multi‐Ethnic Atherosclerosis investigations reported that MAC independently predicted AF development.^25^, ^34^ The Strong Heart Study^35^ reported that left atrium enlargement was key to relationships between AF and MAC. Also, MAC may interrupt inter‐ and intra‐atrial conduction, causing atrial conduction system defects, thus causing AF.^29^ Lewicka et al.^15^ reported that MAC predicted paroxysmal AF recurrence after ablation. We suggest that early AF detection and treatment in patients with MAC should be performed to prevent related stroke, while high conduction system abnormality risks warrant closer monitoring. Thus, MAC may not just be an AF risk factor, but an important prognostic predictor and potential postoperative evaluation index. In patients with MAC, doctors should inaugurate AF preventative measures and reduce adverse outcomes and associated burden if AF is evident. We observed that AF was more common in patients with MAC, consistent with left atrial dilatation.^36^, ^37^ MAC patients susceptible to AF may require rhythm control strategies, while patients with complicated AF may require more rigorous anticoagulation regimens. MAC occurrence should increase suspicion for arrhythmia, thus close postprocedural monitoring is strongly advised in MVD patients with MAC.

Surgical MV repair or replacement is generally considered as the gold standard treatment in patients with established indications.^38^, ^39^ Recently, transcatheter intervention therapy has achieved good safety and efficacy in high‐risk surgical patients.^40^, ^41^ Guerrero et al.^8^ performed transcatheter MV replacement for patients with severe MAC who were not surgical candidates, they found cardiac‐CT based score provided a systematic method to grade MAC severity which may assist in predicting valve embolization/migration. A meta‐analysis showed that the feasibility of transcatheter technology in serious MAC needed to be further explored and improved.^42^ The experience in this aspect is still limited and general recommendations cannot yet be made.^16^ But what is certain is that imaging is critical to the success of these surgical and transcatheter therapies.^43^ Cardiac‐CT can provide the entire mitral valvular and subvalvular structures details (e.g., calcification) before and after operation, it can be an additional important evaluation tool in deciding for the best operation method and evaluating the MV disease pre‐operatively and predicting the prognosis.

### 4.1 Limitations

Our investigation had several limitations. As a retrospective single‐center investigation with a small sample size, selection bias was a possibility. Also, no standard methods categorizing MAC severity using CT have been established.^8^, ^10^ Therefore, larger multicenter studies with larger sample sizes are required to assess quantitative MAC score assessments for predicting disease outcomes.

## 5 CONCLUSIONS

MAC scores from noncontrast cardiac‐gated CT provide clinically important information before valve surgery, and warrant closer monitoring for arrhythmia.

## CONFLICT OF INTEREST STATEMENT

The authors declare no conflict of interest.

## Supporting information

Supporting information.

## Data Availability

The data underlying this article will be shared on reasonable request to the corresponding author.
