# Supplementary material for: Noncontrast cardiac computed tomography‐derived mitral annular calcification scores in mitral valve disease
Source: Clin Cardiol. 2023 Aug 4;46(11):1310–8. doi: 10.1002/clc.24110 (PMC10642324; doi:10.1002/clc.24110)
Supplement: Supplementary file 1 — Supporting information. [file CLC-46-1310-s001.pdf]

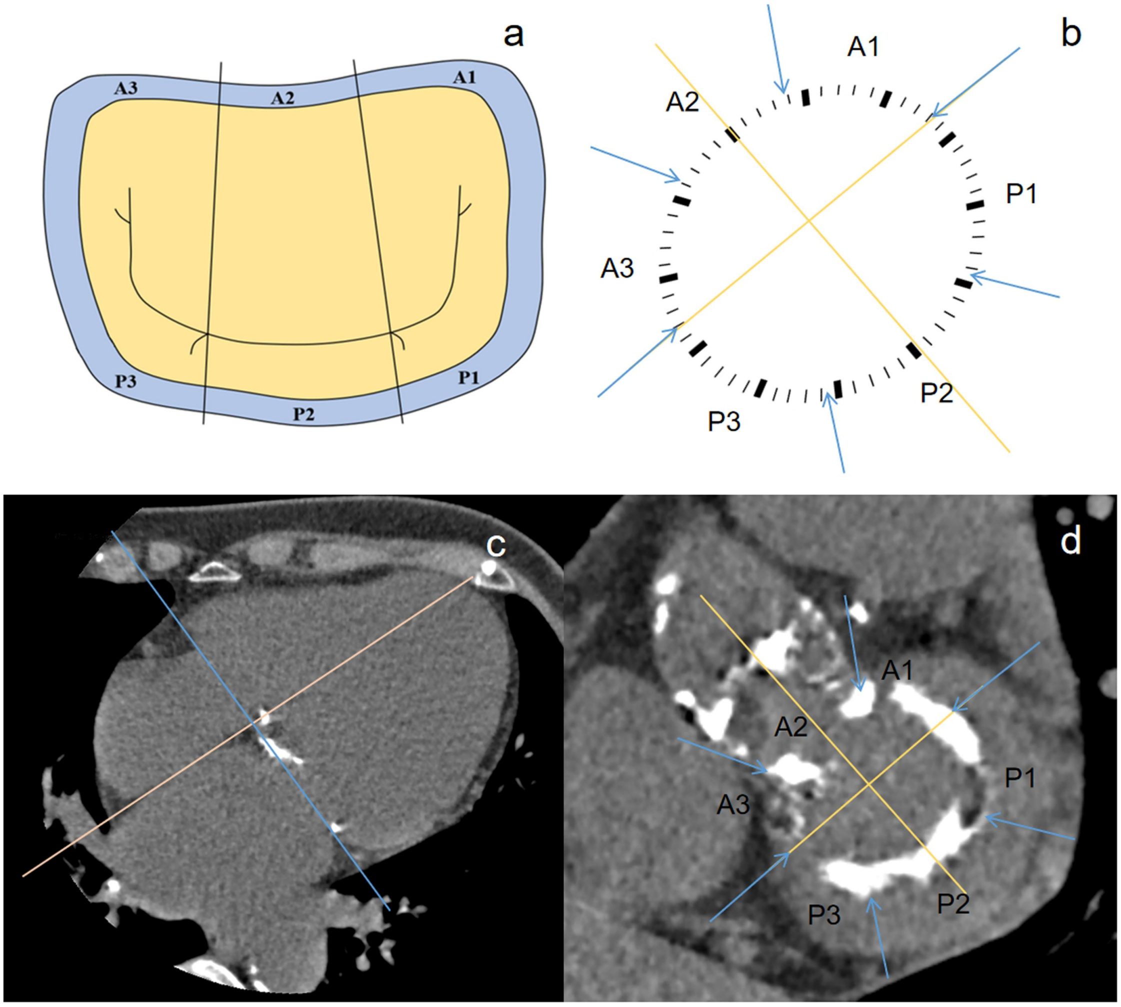

Supplementary Figure 1 Method for localizating mitral annular calcium (MAC) deposits a. The annular segments: A1, A2, A3, segments of the anterior mitral annulus; P1, P2, P3, segments of the posterior mitral annulus, from lateral to medial. **b.** A schematic template of the mitral annulus was created from non-contrast cardiac-gated CT scans, with the annulus divided into 6 segments. A line was drawn through the aortic valve and P2 segment of the mitral annulus. These lines allowed proper positioning of the template. **c.** CT with four-chamber view. **d.** Calcification location was assigned using the template and the Agatston's metod.
